# Adult ADHD, *PARK2* copy number variations and the blood-brain barrier: Culture duration-dependent metabolic alterations in human induced pluripotent stem cell-derived in vitro models

**DOI:** 10.1186/s12987-026-00892-5

**Published:** 2026-10-24

**Authors:** Markus Glaser, Liane Dieterich, Laura Ceslik, Zora Schickardt, Sabine Gätzner, Luisa Schmitt, Heike Oberwinkler, Jasmin Reining, Mike Friedrich, Katherina Hemmen, Werner Schmitz, Rhiannon Victoria McNeill, Sarah Kittel-Schneider, Antje Appelt-Menzel

**Affiliations:** 1 Department for Functional Materials in Medicine and Dentistry, University Hospital Würzburg, Würzburg, Germany; 2 Department of Psychiatry, Psychosomatics and Psychotherapy, University Hospital Würzburg, Würzburg, Germany; 3 Department of Psychiatry and Psychotherapy, University Hospital Erlangen, Friedrich-Alexander University Erlangen-Nuremberg, Erlangen, Germany; 4 Rudolf Virchow Center for Integrative and Translational Bioimaging, University of Würzburg, Würzburg, Germany; 5 Department of Biochemistry and Molecular Biology, University of Würzburg, Würzburg, Germany; 6 Department of Psychiatry and Neurobehavioural Science, University College Cork, Cork, Ireland; 7 Translational Center Regenerative Therapies (TLC-RT), Fraunhofer Institute for Silicate Research ISC, Würzburg, Germany

**Keywords:** Attention-deficit/hyperactivity disorder, PARK2, Copy number variation, Mitochondrial dysfunction, Blood-brain barrier, Endothelial cells, Induced pluripotent stem cells, Mitochondrial membrane potential, Reactive oxygen species, Cellular bioenergetics

## Abstract

**Background:**

Attention-deficit/hyperactivity disorder (ADHD) is a multifactorial neurodevelopmental disorder in which *PARK2* copy number variations (CNVs) constitute a rare genetic risk factor. *PARK2* encodes parkin, a key regulator of mitochondrial quality control. Mitochondrial dysfunction is implicated in ADHD pathophysiology and can contribute to blood-brain barrier (BBB) disruption. As BBB disruption remains insufficiently understood in ADHD, this study investigated whether adult ADHD and *PARK2* CNVs are accompanied by alterations in BBB phenotype, mitochondrial properties, and cellular metabolism.

**Methods:**

Brain capillary endothelial-like cells (BCECs) were generated using 10 donor-specific human induced pluripotent stem cell (hiPSC) lines from adults with ADHD carrying *PARK2* CNVs, alongside *PARK2* wild type adults with and without ADHD. BCECs were analyzed at the end of differentiation (day 10) and after prolonged culture (day 17) to assess group-associated phenotypes and culture duration-dependent differences.

**Results:**

Across all groups, BCECs showed brain endothelial marker expression, high transendothelial electrical resistance (TEER), and low sodium fluorescein (NaFl) permeability at day 10. After prolonged culture, NaFl permeability remained low, whereas TEER-based barrier integrity declined. Prolonged culture of BCECs reduced glycolytic ATP production rates while increasing mitochondrial ATP production and its relative contribution to total ATP production, reflecting a metabolic shift from glycolysis toward oxidative phosphorylation. Despite decreased total ATP production after prolonged culture, cellular energy status was largely preserved, alongside overall lower intracellular reactive oxygen species (ROS) and mitochondrial membrane potential. At day 10, BCECs from ADHD donors carrying *PARK2* duplications displayed greater mitochondrial abundance, the highest basal and oxidative stress-induced intracellular ROS levels, and reduced TEER-based barrier integrity, while at day 17, BCECs from ADHD donors with *PARK2* deletions exhibited the least glycolysis-dependent ATP production profile, although absolute mitochondrial ATP production remained comparable between groups.

**Conclusions:**

Prolonged culture was associated with broad, ADHD- and *PARK2* genotype-independent metabolic remodeling, whereas mitochondrial and metabolic alterations associated with ADHD and *PARK2* CNVs were overall small. This study identifies culture duration as a critical parameter for the characterization of hiPSC-derived BBB in vitro models and supports the feasibility of using donor-specific BCECs at the end of differentiation to investigate BBB-associated mechanisms in adult ADHD and *PARK2* CNVs.

**Supplementary Information:**

The online version contains supplementary material available at https://doi.org/10.1186/s12987-026-00892-5.

## Background

Attention-deficit/hyperactivity disorder (ADHD) is among the most prevalent neurodevelopmental disorders worldwide and is characterized by age-inappropriate levels of inattention, hyperactivity, and impulsivity that interfere with academic, occupational, and social functioning [1–3]. Symptoms usually begin in childhood and may persist into adult life, with epidemiological studies reporting a prevalence of 5.3% in school-aged children and about 2.5% in adults [4, 5]. ADHD is highly heritable, and genetic predisposition accounts for a substantial proportion of interindividual variation in liability, while environmental risk factors across prenatal, perinatal, and psychosocial domains also contribute to its development, supporting a multifactorial etiology [2, 6]. Recent genome-wide association studies have identified multiple common genetic variants linked to ADHD, predominantly single nucleotide polymorphisms, and have enabled follow-up analyses that map these loci to candidate genes and biological pathways [7, 8]. Beyond this overall polygenic background, rare genomic alterations including copy number variations (CNVs) have also been associated with ADHD and partly overlap with loci implicated in other neurodevelopmental and psychiatric disorders [9]. Deletions and duplications in *PARK2* are observed at higher frequencies in individuals with ADHD than in controls, supporting *PARK2* as a rare structural risk locus for the disorder [10]. While biallelic loss-of-function mutations in *PARK2* cause early-onset Parkinson’s disease, *PARK2* CNVs are thought to induce more subtle disturbances in parkin-mediated mitochondrial quality control [11, 12].

Encoded by *PARK2*, the E3 ubiquitin ligase parkin acts together with PTEN-induced kinase 1 (PINK1) to regulate mitophagy and mitochondrial dynamics, thereby maintaining mitochondrial function, including ATP production and redox homeostasis [13, 14]. In fibroblasts and human induced pluripotent stem cell (hiPSC)-derived dopaminergic neurons from adults with ADHD carrying *PARK2* CNVs, parkin-related mitochondrial dysfunction was characterized by altered mitochondrial network morphology, lower ATP levels, and reduced oxygen consumption rates [12]. Moreover, accumulating clinical and preclinical evidence suggests that mitochondrial dysfunction is involved in ADHD pathophysiology, with reported perturbations in energy metabolism and redox balance [15, 16]. Blood-brain barrier (BBB) disruption is commonly observed across disorders of the central nervous system and can impair brain homeostasis, while recent findings suggest a potential link between ADHD and BBB disruption, although the underlying mechanisms and pathophysiological implications remain unclear [17–19]. Brain endothelial cells rely on intact mitochondrial function to maintain tight junction-dependent barrier integrity, support active transport across the endothelium, and limit oxidative stress that would otherwise compromise barrier properties [20, 21]. Reduced parkin expression in brain endothelial cells was associated with impaired mitophagy and mitochondrial damage, while mitochondrial dysfunction has been linked to BBB disruption, accompanied by elevated levels of reactive oxygen species (ROS), tight junction disorganization, increased permeability, and altered transport processes [22, 23].

ADHD is most often treated pharmacologically, with stimulant medications as first-line therapy and several non-stimulant agents available as alternatives, all aimed at reducing core symptoms and functional impairment rather than targeting causal pathomechanisms [24, 25]. Adult ADHD frequently remains underdiagnosed, and even when pharmacotherapy is initiated, up to 50% of adult patients respond insufficiently to medication, in part due to comorbid psychiatric and somatic disorders that further limit treatment effectiveness [26–28]. Therefore, adult ADHD patient-specific in vitro models are required to better understand disease mechanisms in individual patients and to provide a translational platform for testing and refining treatment strategies. In addition, such models could enable evaluation of potential contributions of BBB disruption to pathophysiology and therapeutic response.

The aim of this study was to examine whether adult ADHD and *PARK2* CNVs are associated with alterations in BBB phenotype, mitochondrial properties, and broader cellular metabolism. For this purpose, BBB in vitro models based on brain capillary endothelial-like cells (BCECs) were generated from hiPSCs derived from adult ADHD patients carrying *PARK2* CNVs and control donors. Accordingly, models were analyzed at the end of differentiation and after prolonged in vitro culture to evaluate both group-associated phenotypes and culture duration-dependent alterations.

## Methods

### Derivation and maintenance of hiPSCs

Donor-specific hiPSC lines were generated and provided by the Department of Psychiatry, Psychosomatics and Psychotherapy, University Hospital Würzburg, using the CytoTune™-iPS 2.0 Sendai Reprogramming Kit (Thermo Fisher Scientific, A16517) according to the manufacturer’s instructions. For quality control, hiPSCs were assessed for pluripotency, trilineage differentiation, and Sendai virus clearance (Fig. S1), and additional mycoplasma and genetic analyses were performed as previously described [12, 29–31]. Donor and hiPSC line characteristics of the 4 WT_CTRL, 2 WT_ADHD, 2 DEL_ADHD, and 2 DUP_ADHD hiPSC lines are summarized in Table S1.

HiPSCs were cultivated on 6-well plates (Thermo Fisher Scientific, 140675) coated with 83.3 µg/mL Matrigel^®^ (Corning, 356231) at 37 °C, 5% CO_2_, and 95% humidity. Twice per week at around 80% confluency, cells were passaged with ReLeSR™ (STEMCELL Technologies, 100–0483) and reseeded at ratios of 1:8 to 1:20 in StemMACS™ PSC-Brew XF medium (Miltenyi Biotec, 130-127-865) supplemented with 10 µM Y-27632 (Tocris Bioscience, 1254/10). Medium was replaced 24 h after seeding, and the volume was doubled on Fridays to allow weekend-free culture.

### Generation of hiPSC-derived BCECs

For differentiation of hiPSCs into BCECs, the co-differentiation protocol of Lippmann et al. was applied with slight modifications [32]. At day −3, hiPSCs were detached with Accutase^®^ (Sigma-Aldrich, A6964-100ML), resuspended in StemMACS™ PSC-Brew XF medium with 10 µM Y-27632, and seeded at a density of 7.8 × 10^3^ cells/cm^2^ on Matrigel^®^-coated 6-well plates. Between day 0 and day 5, daily medium changes were performed with unconditioned medium containing 78.5% DMEM/F-12 without glutamine (Thermo Fisher Scientific, 21331020), 20.0% KnockOut™ Serum Replacement (Thermo Fisher Scientific, 10828028), 1.0% MEM non-essential amino acids (Thermo Fisher Scientific, 11140035), 1 mM L-glutamine (Sigma-Aldrich, G7513-100ML), and 0.1 mM 2-mercaptoethanol (Thermo Fisher Scientific, 31350010). At day 6 of differentiation, medium was changed to EC medium containing 99.5% human endothelial SFM (Thermo Fisher Scientific, 11111044) and 0.5% B-27™ without vitamin A (Thermo Fisher Scientific, 12587010), supplemented with 20 ng/mL hbFGF (Thermo Fisher Scientific, 100-18B-500UG) and 10 µM retinoic acid (Miltenyi Biotec, 130-117-339). At day 8, cells were detached with Accutase^®^, resuspended in EC medium supplemented with 20 ng/mL hbFGF and 10 µM retinoic acid, and seeded at a density of 1.0 × 10^6^ cells/cm^2^ on 24-well inserts (cellQART^®^, 9320412), 8-well Nunc™ Lab-Tek™ chamber slides (Thermo Fisher Scientific, 177445PK), 96-well plates (Greiner Bio-One, 655090; Agilent, 103774-100), and 12-well plates (TPP^®^, 92012), all coated with 200 µg/mL Matrigel^®^. Between day 9 and day 17, EC medium was replaced daily on the apical side and at day 9, day 10, day 14, and day 17 in the basolateral compartment.

### Transendothelial electrical resistance measurement

Transendothelial electrical resistance (TEER) values of BCECs at day 10 and day 17 were determined using a Millicell^®^ ERS-2 V-ohmmeter (Merck) equipped with a STX3 electrode (World Precision Instruments). Prior to usage, the electrode was equilibrated in EC medium for 15 min. Measurements were initiated 45 min after medium change on a heating plate maintained at 37 °C. Blank values for Matrigel^®^-coated inserts without cells were calculated as the mean of three measurements. For each independent differentiation experiment, electrical resistance of at least 3 inserts containing BCECs was measured in duplicate or triplicate and corrected by subtracting blanks. Results were multiplied by the culture area of a 24-well insert.

### Sodium fluorescein permeability assay

Sodium fluorescein (NaFl) permeability of BCECs cultured on 24-well inserts was assessed at day 10 and day 17. For each independent differentiation experiment, three technical replicate inserts were prepared, with Matrigel^®^-coated cell-free inserts serving as blanks. EC medium supplemented with 10 µM NaFl (Sigma-Aldrich, F6377-100G) was applied apically, while EC medium without NaFl was added to the basolateral compartment. Inserts were incubated on an orbital shaker at 100 rpm for 60 min at 37 °C. Apical and basolateral samples, together with EC medium containing 10 µM NaFl and EC medium without NaFl, were transferred to 96-well plates (Greiner Bio-One, 655076), and fluorescence intensity was measured at 490 nm excitation and 525 nm emission using an Infinite^®^ M200 microplate reader (Tecan). Apical fluorescence values were adjusted for dilution with EC medium. As described previously [33], the permeability coefficient (PC) was calculated from NaFl clearance after 60 min, corrected for NaFl passage through cell-free inserts, and divided by insert surface area.

### Immunofluorescence staining

At day 10 and day 17, BCECs cultured on inserts or 8-well chamber slides were fixed with 4.0% paraformaldehyde (AppliChem, A3813.1000) for 10 min, washed with PBS (Sigma-Aldrich, D8537-500ML), and permeabilized with 0.2% Triton^®^ X-100 (Carl Roth, 3051.2) for 5 min. Cells were washed with 0.5% PBST (Sigma-Aldrich, P1379-25ML) and blocked in blocking buffer containing 5.0% donkey serum (Biozol Diagnostica, ECL-ECS0217L), 0.1% Triton^®^ X-100, and 0.02% saponin (Carl Roth, 9622.1) for 20 min. The primary antibodies vascular endothelial cadherin (CDH5, Sigma-Aldrich, V1514-200UL, 1:100), glucose transporter 1 (GLUT1, Abcam, ab115730, 1:100), occludin (OCLN, Thermo Fisher Scientific, 33-1500, 1:100), claudin-5 (CLDN5, Abcam, ab15106, 1:100), and translocase of the outer mitochondrial membrane 20 (TOM20, Proteintech, 66777-1-Ig, 1:400) were diluted in blocking buffer and incubated overnight at 4 °C. Samples were washed with PBST and incubated with Alexa Fluor™ 488-, 555-, or 647-conjugated secondary antibodies (Thermo Fisher Scientific, A-21202; A-31570; A-31573, 1:400) diluted in antibody diluent (DCS Diagnostics, AL120R500) for 60 min. BCECs were washed, membranes were excised from inserts, and mounted on object slides using Fluoromount-G™ Mounting Medium with DAPI (Thermo Fisher Scientific, 00-4959-52). Images were acquired using the Quick Full Focus function on a BZ-X810 fluorescence microscope (Keyence) with a 20× objective (NA 0.75) for qualitative assessment of CDH5, GLUT1, OCLN, and CLDN5.

### Quantification of mitochondrial morphology

TOM20- (Alexa Fluor™ 555) and DAPI-stained BCECs were imaged using an LSM 980 confocal microscope (Zeiss) with a 40× water immersion objective (NA 1.20). For each differentiation experiment, 5 fields of view were recorded. Z-stacks were deconvolved in Huygens (Scientific Volume Imaging) using Classic Maximum Likelihood Estimation with a theoretical point spread function. Maximum intensity projections were generated in ImageJ (National Institutes of Health) and batch-processed with the Mitochondria Analyzer plugin [34] to quantify mitochondrial count, area, aspect ratio, and form factor. Pre-processing included a sigma filter plus with radius 2.00, enhancement of local contrast with maximum slope 1.80, and gamma adjustment to 0.80. Local thresholding used the weighted mean method with a block size of 1.250 microns and a C-value of 10. For post-processing, despeckle was applied. Aspect ratio was defined as the ratio of the major to minor axis, and form factor was calculated as perimeter squared divided by the product of 4π and area. Values equal to 1 for both aspect ratio and form factor indicate mitochondria without elongation and a perimeter consistent with a circular shape for the measured area, whereas higher values reflect increased elongation and greater deviation from a circle. Cell counts per image were obtained from DAPI-stained nuclei using Cellpose [35].

### Mitochondrial membrane potential measurement

Mitochondrial membrane potential (ΔΨm) of BCECs at day 10 and day 17 was assessed in 96-well plates using tetramethylrhodamine methyl ester (TMRM) staining, based on a publication by Zink et al. with minor adaptations [36]. Briefly, cells were incubated for 30 min in EC medium supplemented with 150 nM TMRM (Thermo Fisher Scientific, T668) and 2 µg/mL Hoechst 33342 (Thermo Fisher Scientific, H3570), together with either dimethyl sulfoxide (Sigma-Aldrich, D8418-500ML) as vehicle control or 20 µM carbonyl cyanide-p-trifluoromethoxyphenylhydrazone (FCCP, Sigma-Aldrich, C2920-10MG) plus 20 µM antimycin A (AA, Sigma-Aldrich, A8674-25MG) to induce mitochondrial depolarization. For each differentiation experiment, cells were seeded in triplicate for both conditions, and 3 fields of view were recorded per well. Images were acquired using a CellVoyager™ CQ1 confocal quantitative image cytometer (Yokogawa) with a 20× long working distance objective (NA 0.45), using Hoechst 33342 for nuclear staining. As described by Zink et al. [36], ΔΨm was quantified using CellProfiler [37].

### ROS assay

Intracellular ROS levels in BCECs were determined at day 10 and day 17 in 96-well plates. For ROS assays, EC medium contained 0.5% B-27™ without antioxidants (Thermo Fisher Scientific, 10889038) instead of B-27™ without vitamin A. Cells were incubated for 30 min in EC medium with 20 µM 2′,7′-dichlorodihydrofluorescein diacetate (DCFDA, Sigma-Aldrich, D6883-50MG) in the presence or absence of 1 mM *tert*-butyl hydroperoxide (TBHP, Sigma-Aldrich, 458139-25ML). For each differentiation experiment, BCECs were seeded in triplicate for both conditions. Plates were rinsed with PBS, and fluorescence intensity was measured in PBS at 485 nm excitation and 535 nm emission with an Infinite^®^ M200 microplate reader (Tecan). Fluorescence intensity values were corrected by subtracting blank signals from DCFDA-treated wells, with or without TBHP.

After measurements, BCECs were fixed with 4.0% paraformaldehyde for 10 min, washed with PBS and stained with 0.2 µg/mL DAPI (Thermo Fisher Scientific, 62248) for 10 min. Plates were washed with PBS and imaged on a CellVoyager™ CQ1 confocal quantitative image cytometer with a 20× long working distance objective (NA 0.45), acquiring z-stacks from 5 fields of view per well. Mean cell count per field of view was quantified in CellPathfinder (Yokogawa) and scaled to the total well area.

### Seahorse XF analysis

Seahorse XF Real-Time ATP Rate Assays (Agilent, 103592-100) were performed with BCECs at day 10 and day 17 in 96-well plates using a Seahorse XF Pro Analyzer (Agilent). For each differentiation experiment, up to 8 technical replicate wells were prepared. Cells were washed once and incubated with Seahorse XF assay medium containing Seahorse XF DMEM (Agilent, 103575-100), supplemented with 10 mM glucose (Agilent, 103577-100), 1 mM pyruvate (Agilent, 103578-100), and 2 mM glutamine (Agilent, 103579-100) for 60 min at 37 °C in a non-CO_2_ incubator. According to the manufacturer’s instructions, BCECs were treated with 1.5 µM oligomycin and 0.5 µM rotenone plus 0.5 µM antimycin A through the injection ports of a hydrated Seahorse XF sensor cartridge (Agilent, 103775-100). Results were analyzed using Seahorse Analytics (Agilent).

After measurements, cell counts per well were determined as described for ROS assays, acquiring only 2 fields of view per well due to smaller well area.

### Quantification of intracellular metabolites

BCECs were cultured in 12-well plates and processed for intracellular metabolite quantification at day 10 and day 17. Cells were rinsed twice with cold 154 mM ammonium acetate (Sigma-Aldrich, A7330-500G) and harvested by cell scraping in a prechilled mixture of 80% v/v methanol (Sigma-Aldrich, 32213–2.5 L-M) and water. Samples were normalized to DNA content using a NanoPhotometer™ N60 (Implen). Liquid chromatography mass spectrometry analysis was performed using an UltiMate 3000 UHPLC system (Thermo Fisher Scientific) connected to a Q Exactive mass spectrometer (Thermo Fisher Scientific) equipped with a heated electrospray ionization source. Briefly, samples were fortified with external standards, ultrasonicated, centrifuged, dried, and reconstituted in 50% v/v acetonitrile (Merck, 1000292500) and water containing 5 mM ammonium acetate. Metabolite separation was performed on a BEH Amide column using a gradient of acetonitrile and water containing ammonium acetate. Data were acquired in alternating positive and negative full-scan mode, and peaks were manually curated and integrated using TraceFinder (Thermo Fisher Scientific).

### Statistical analysis

Analyses were performed using GraphPad Prism (GraphPad Software). Data are reported as mean ± SD, where each independent differentiation experiment constitutes a biological replicate. Outliers were identified with the ROUT method (Q = 1%). Normal distribution of data was assessed by the Shapiro-Wilk test. For comparisons of more than two independent data sets, one-way ANOVA followed by Tukey’s post hoc test was performed when parametric assumptions were met. Otherwise, the Kruskal-Wallis with Dunn’s post hoc test was applied. For analyses involving group and either culture duration or treatment, two-way ANOVA followed by Sidak’s post hoc test was used to compare groups within each culture duration or treatment condition and to assess prolonged culture- or treatment-dependent effects within the same group. For comparisons of two independent data sets, pairwise comparisons were performed by Mann-Whitney or unpaired Welch’s t-tests. Statistical significance was defined as: *p < 0.05, **p < 0.01, ***p < 0.001, ****p < 0.0001.

## Results

### ADHD *PARK2* CNV-derived BCECs preserve cellular identity and establish functional barrier properties at day 10

In this study, BCECs were derived from 10 donor-specific hiPSC lines across 4 different groups: (I) *PARK2* wild type adults without ADHD (WT_CTRL), (II) *PARK2* wild type adults with ADHD (WT_ADHD), (III) *PARK2* deletion carriers with adult ADHD (DEL_ADHD), and (IV) *PARK2* duplication carriers with adult ADHD (DUP_ADHD). BCECs were analyzed at the end of differentiation (day 10) and after 7 days of additional culture (day 17), with each assay performed at both time points to investigate alterations associated with culture duration.

**Fig. 1:**
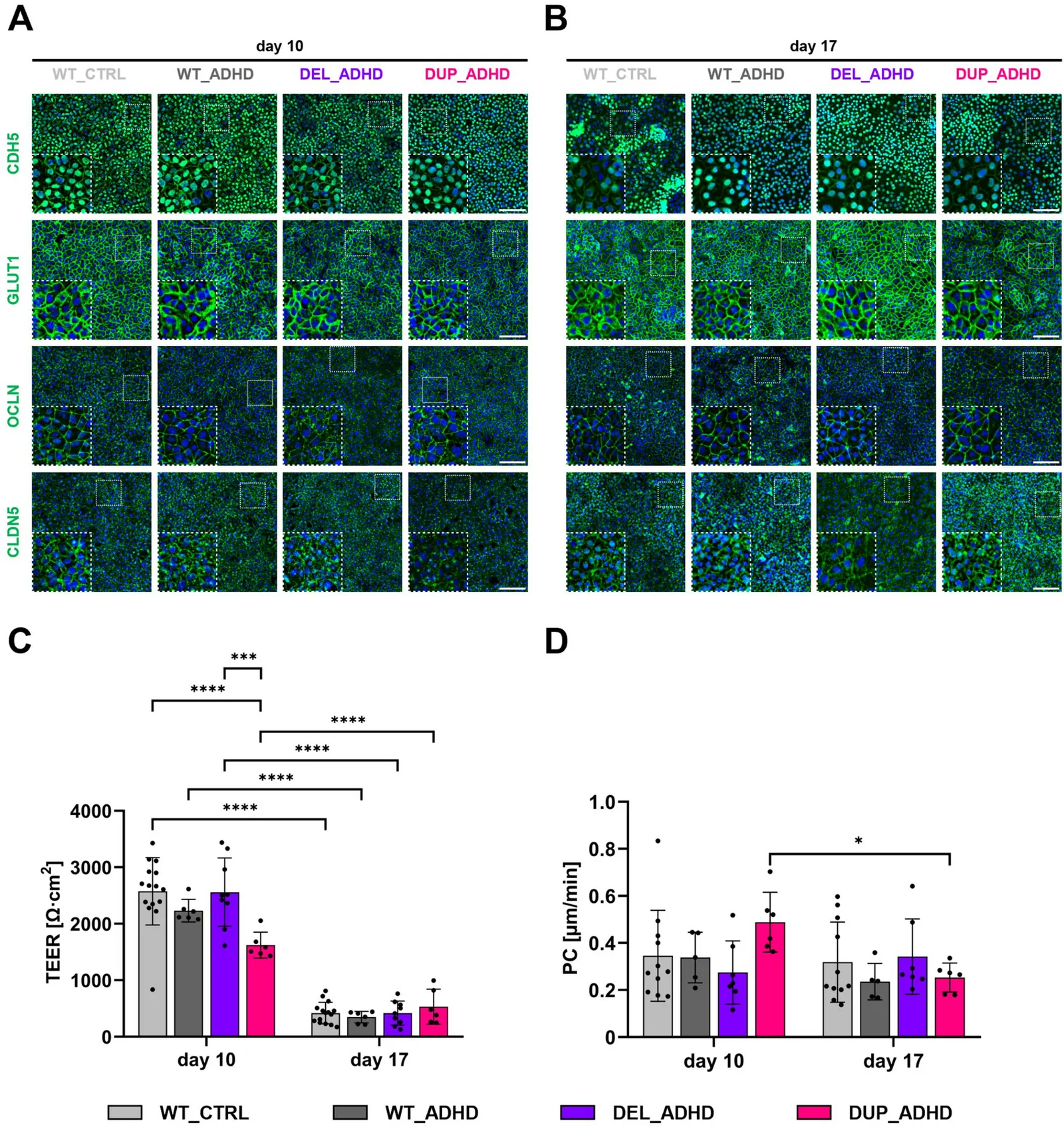
ADHD *PARK2* CNV-derived BCECs maintained cellular identity and achieved functional barrier tightness at day 10. (**A**–**B**) Representative immunofluorescence images of BCECs at day 10 (**A**) and day 17 (**B**) derived from donor-specific hiPSC lines WT_CTRL (#1), WT_ADHD (#1), DEL_ADHD (#1), and DUP_ADHD (#1). BCECs were stained for CDH5, GLUT1, OCLN, and CLDN5 (green), followed by DAPI counterstaining (blue). Scale bars, 100 μm. (**C**–**D**) Barrier integrity of BCECs at day 10 and day 17, determined by TEER measurements (**C**) and NaFl permeability assay (**D**). Data are shown as mean ± SD from *n* ≥ 6 (C), *n* ≥ 5 (**D**) independent differentiation experiments, each representing a biological replicate. Statistical analysis was performed using two-way ANOVA with Sidak’s post hoc test (**C**, **D**): *p < 0.05, ***p < 0.001, ****p < 0.0001

To evaluate the capacity of each patient-derived hiPSC line to differentiate into BCECs with brain endothelial identity, we performed immunofluorescence staining for characteristic markers. At day 10 of differentiation, BCECs expressed the endothelial cell marker CDH5, showed plasma membrane enrichment of GLUT1, and displayed the tight junction proteins OCLN and CLDN5 at cell-cell boundaries (Fig. 1A). No apparent differences in marker expression or localization were detected across all groups at day 10, suggesting preserved cellular identity of BCECs independent of *PARK2* genotype and ADHD. At day 17, BCECs maintained cell type-specific markers (Fig. 1B), indicating that the qualitative marker profile was not altered by the duration of in vitro culture.

Furthermore, TEER and NaFl permeability were assessed to determine whether *PARK2* CNVs and ADHD influence barrier properties of BCECs. At day 10, TEER was significantly reduced in DUP_ADHD BCECs compared with WT_CTRL and DEL_ADHD BCECs (Fig. 1C), although all groups established functional barriers with mean TEER values above 1600 Ω cm^2^. TEER did not differ significantly between groups at day 17, whereas each group showed significantly diminished TEER after prolonged culture, resulting in mean values below 550 Ω cm^2^. Mean NaFl permeability remained below 0.5 μm/min and comparable across groups at day 10 and day 17 (Fig. 1D), while DUP_ADHD BCECs had the numerically highest mean PC at day 10. After prolonged culture, PCs were similar within WT_CTRL, WT_ADHD, and DEL_ADHD BCECs, whereas DUP_ADHD BCECs displayed a significant decrease, indicating improved paracellular barrier function. Taken together, ADHD *PARK2* CNV-derived BCECs retained cellular identity and formed physiologically tight barriers at day 10, whereas prolonged culture reduced TEER-based barrier integrity in all groups without increasing NaFl permeability.

### Mitochondrial morphology remains intact in ADHD *PARK2* CNV-derived BCECs

**Fig. 2:**
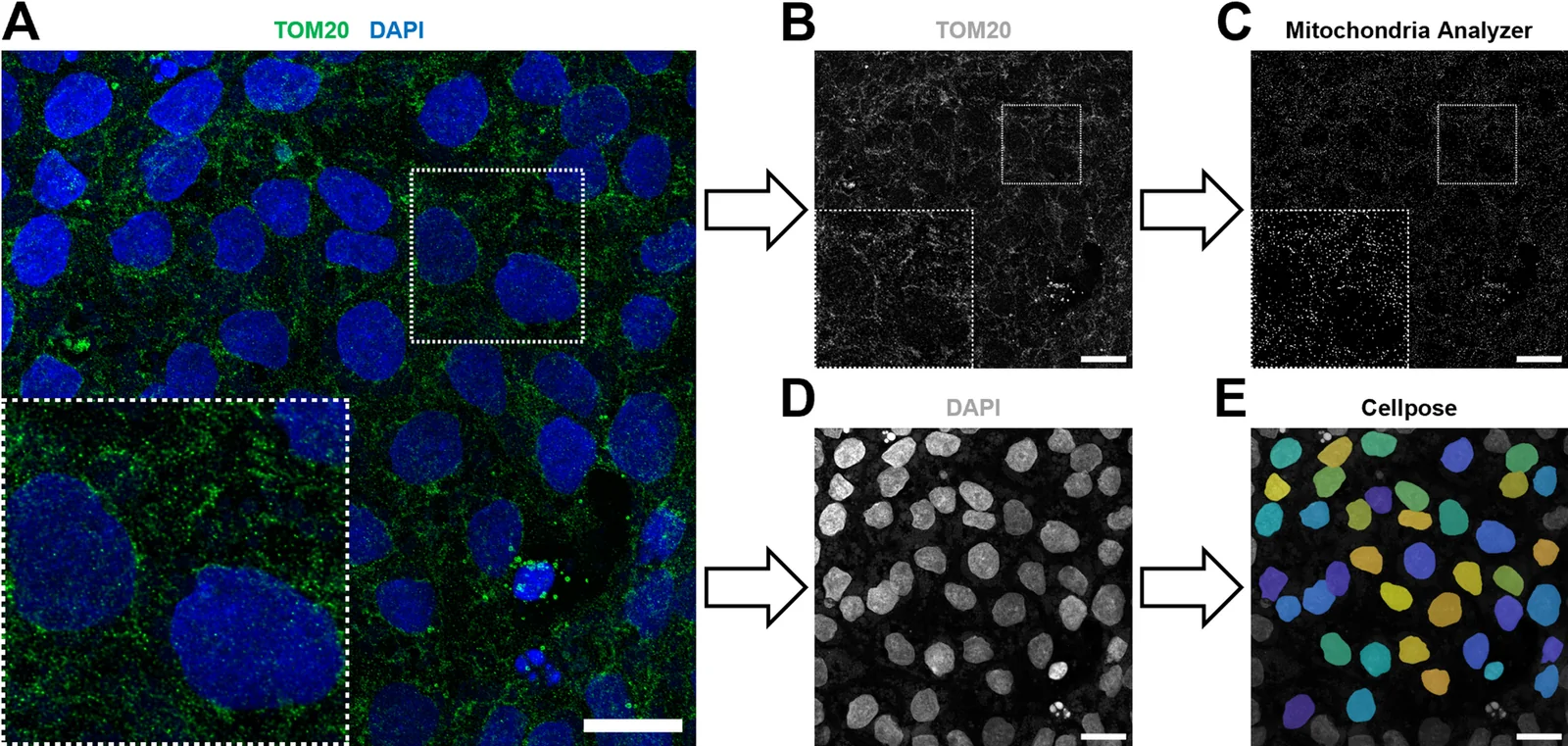
Schematic workflow of the image analysis to quantify mitochondrial abundance and morphology. (**A**) BCECs were stained for TOM20 (green), followed by DAPI counterstaining (blue). Scale bars, 20 μm. (**B**–**E**) TOM20 (**B**) and DAPI (**D**) channels were separated, and segmentation masks were generated using Mitochondria Analyzer (**C**) for mitochondria and Cellpose (**E**) for nuclei, respectively. Nuclear counts were used to normalize mitochondrial counts to cell number

Following successful establishment of donor-specific BBB in vitro models and given the role of parkin in mitochondrial quality control, we next analyzed whether mitochondrial morphology in BCECs varied with *PARK2* CNVs and ADHD. Immunofluorescence staining for TOM20 was performed in BCECs to quantify mitochondrial abundance and morphology using Mitochondria Analyzer (Fig. 2). Mitochondrial count, area, aspect ratio, and form factor were quantified in BCECs at day 10 and day 17. Figure S2 provides representative images of TOM20-stained BCECs at day 10 and day 17 from the dataset used for quantification. At day 10, mitochondrial count was significantly increased in DUP_ADHD BCECs compared with WT_CTRL, followed by a significant decrease within DUP_ADHD BCECs after prolonged culture (Table 1). Area per mitochondrion was consistent across all groups at day 10, at day 17, and within BCECs after prolonged culture. Mitochondrial aspect ratio and form factor were similar between groups at both day 10 and day 17. After prolonged culture, aspect ratio and form factor were slightly reduced in every group, with significant reductions observed only in WT_CTRL and DEL_ADHD BCECs. In conclusion, ADHD *PARK2* CNV-derived BCECs exhibited intact mitochondrial morphology at both day 10 and day 17, while aspect ratio and form factor decreased in WT_CTRL and DEL_ADHD BCECs after prolonged culture.

**Table 1:** ADHD *PARK2* CNV-derived BCECs preserved mitochondrial morphology

	WT_CTRL	WT_ADHD	DEL_ADHD	DUP_ADHD
**Day 10**				
Mitochondrial count per cell	86.94 ± 26.94^a)^	105.20 ± 9.42	112.39 ± 35.08	161.62 ± 66.00^a), b)^
Area per mitochondrion [µm^2^]	0.092 ± 0.007	0.094 ± 0.005	0.106 ± 0.017	0.100 ± 0.011
Aspect ratio per mitochondrion	1.64 ± 0.13^c)^	1.66 ± 0.08	1.69 ± 0.11^d)^	1.68 ± 0.06
Form factor per mitochondrion	1.16 ± 0.07^e)^	1.16 ± 0.04	1.20 ± 0.07^f)^	1.18 ± 0.04
**Day 17**				
Mitochondrial count per cell	94.77 ± 48.44	69.38 ± 22.72	99.92 ± 40.75	62.47 ± 20.93^b)^
Area per mitochondrion [µm^2^]	0.088 ± 0.003	0.090 ± 0.009	0.096 ± 0.007	0.094 ± 0.006
Aspect ratio per mitochondrion	1.51 ± 0.03^c)^	1.50 ± 0.07	1.53 ± 0.05^d)^	1.53 ± 0.08
Form factor per mitochondrion	1.09 ± 0.01^e)^	1.10 ± 0.03	1.10 ± 0.02^f)^	1.11 ± 0.03

Image-based quantification of mitochondrial count, area, aspect ratio, and form factor in TOM20-stained BCECs at day 10 and day 17. Data are shown as mean ± SD from WT_CTRL n ≥ 8, WT_ADHD n = 4, DEL_ADHD n = 6, DUP_ADHD n ≥ 5 independent differentiation experiments, each representing a biological replicate. ^(a–f)^ Statistical analysis was performed using two-way ANOVA with Sidak’s post hoc test: *p < 0.05 (c, d, e), **p < 0.01 (a, b, f)

### Mitochondrial membrane potential is not impaired in ADHD *PARK2* CNV-derived BCECs, whereas oxidative stress sensitivity is increased in DUP_ADHD BCECs at day 10

**Fig. 3:**
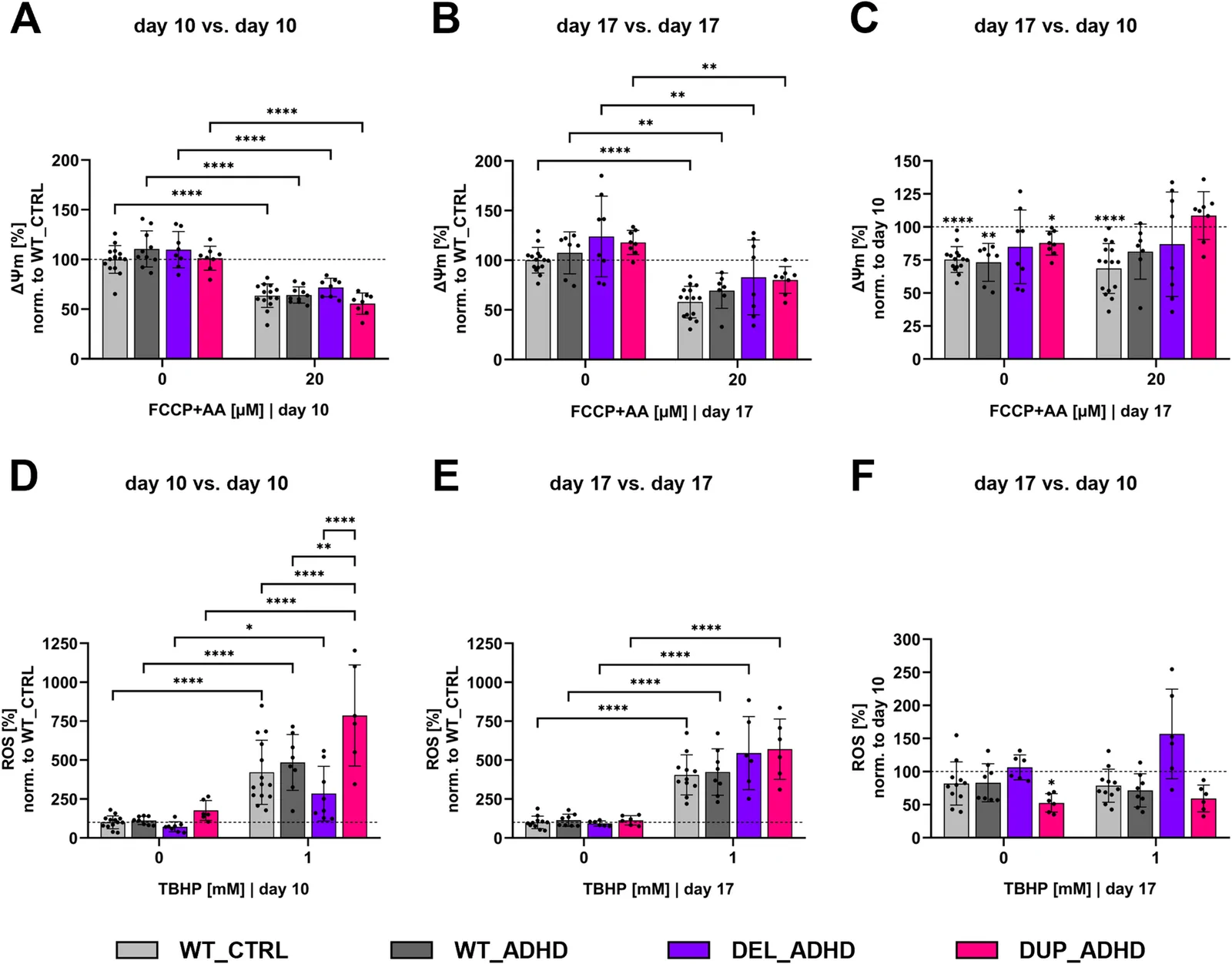
ADHD *PARK2* CNV-derived BCECs retained ΔΨm, with increased DUP_ADHD oxidative stress sensitivity at day 10. (**A**–**C**) ΔΨm of BCECs at day 10 normalized to WT_CTRL (**A**), at day 17 normalized to WT_CTRL (**B**), and at day 17 normalized to day 10 (**C**), determined by TMRM staining. Dashed lines indicate respective 100% reference levels. Cells were treated with 20 µM FCCP and 20 µM AA to assess mitochondrial depolarization. (**D**–**F**) ROS levels of BCECs at day 10 normalized to WT_CTRL (**D**), at day 17 normalized to WT_CTRL (**E**), and at day 17 normalized to day 10 (**F**), determined by ROS assay. Dashed lines indicate respective 100% reference levels. Cells were treated with 1 mM TBHP to induce oxidative stress. Data are shown as mean ± SD from *n* ≥ 7 (**A**–**C**), *n* ≥ 6 (**D**–**F**) independent differentiation experiments, each representing a biological replicate. Statistical analysis was performed using two-way ANOVA with Sidak’s post hoc test (**A**, **B**, **D**, **E**), and pairwise comparisons by Mann-Whitney or unpaired Welch’s t-tests (**C**, **F**): *p < 0.05, **p < 0.01, ****p < 0.0001

Since parkin has been proposed to contribute to ADHD pathophysiology through impaired mitochondrial quality control, we analyzed whether BCECs derived from ADHD patients carrying *PARK2* CNVs displayed alterations in mitochondrial membrane potential (ΔΨm). ΔΨm is a sensitive readout of mitochondrial bioenergetic state and can provide insights into mitochondrial function. Accordingly, we quantified ΔΨm using TMRM under basal conditions and after mitochondrial depolarization with FCCP in combination with AA. Across all groups, basal ΔΨm was comparable in BCECs at day 10 (Fig. 3A) and day 17 (Fig. 3B), whereas FCCP + AA significantly reduced ΔΨm in every group at both time points. Comparing day 17 to day 10 cultures within the same group, ΔΨm showed a significant reduction in WT_CTRL, WT_ADHD, and DUP_ADHD BCECs under basal conditions, and in WT_CTRL BCECs following FCCP + AA treatment (Fig. 3C).

To determine whether *PARK2* CNVs and ADHD were associated with altered oxidative stress, which may arise from mitochondrial dysfunction and contribute to cellular damage, we quantified intracellular ROS in BCECs. ROS levels were measured with and without the oxidative stressor TBHP. At day 10, baseline ROS levels in BCECs did not differ significantly between groups, with a tendency toward higher levels in DUP_ADHD BCECs, whereas TBHP treatment significantly increased ROS in every group (Fig. 3D). Notably, TBHP-treated DUP_ADHD BCECs displayed significantly higher ROS levels compared to every other treated group at day 10. Across all groups, day 17 BCECs showed similar basal ROS, while TBHP treatment again significantly elevated ROS (Fig. 3E). Compared with BCECs at day 10, ROS levels of day 17 BCECs were lower in WT_CTRL, WT_ADHD, and DUP_ADHD, yet higher in DEL_ADHD under both basal and TBHP-treated conditions, with only the decrease in untreated DUP_ADHD BCECs achieving statistical significance (Fig. 3F). Overall, ADHD *PARK2* CNV-derived BCECs did not exhibit perturbations in ΔΨm at either day 10 and day 17, while DUP_ADHD BCECs showed slightly higher basal ROS levels and a greater TBHP-induced ROS response at day 10, indicating heightened sensitivity to oxidative stressors.

### Prolonged culture shifts BCEC metabolism from glycolysis toward oxidative phosphorylation

**Fig. 4:**
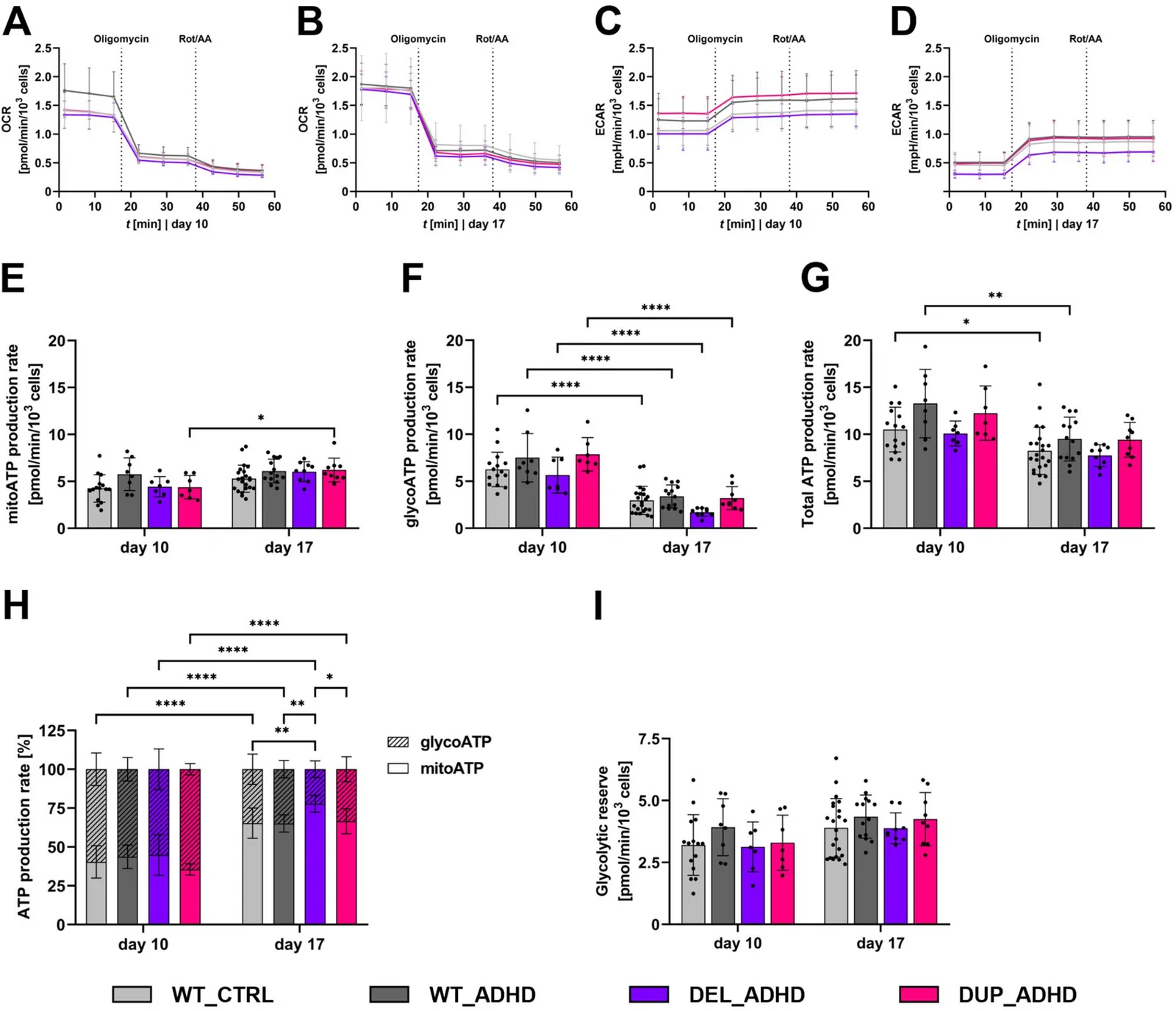
Prolonged culture of BCECs induced a metabolic shift from glycolysis toward OXPHOS within every group. (**A**–**B**) OCR profiles of BCECs at day 10 (**A**) and day 17 (**B**), determined by Seahorse XF Real-Time ATP Rate Assay. (**C**–**D**) ECAR profiles of BCECs at day 10 (**C**) and day 17 (**D**). (**E**–**G**) MitoATP (**E**), glycoATP (**F**), and total ATP (**G**) production rates of BCECs at day 10 and day 17. (**H**) Relative contributions of mitoATP and glycoATP to the total ATP production rate of BCECs at day 10 and day 17. (**I**) Glycolytic reserve of BCECs at day 10 and day 17, calculated as the difference between compensatory glycolysis and basal glycolysis. Data are shown as mean ± SD from *n* ≥ 7 independent differentiation experiments, each representing a biological replicate. Statistical analysis was performed using two-way ANOVA with Sidak’s post hoc test (**E**–**I**): *p < 0.05, **p < 0.01, ****p < 0.0001

Given the overall reduction in ΔΨm and ROS after prolonged culture, we next investigated whether culture duration of BCECs was associated with changes in ATP-generating pathways. As ATP is produced primarily through oxidative phosphorylation (OXPHOS) and glycolysis, mitochondrial (mitoATP) and glycolytic (glycoATP) ATP production rates were quantified in BCECs at day 10 and day 17 by performing Seahorse XF analysis. Oxygen consumption rate (OCR) profiles of BCECs were largely comparable across groups at day 10 (Fig. 4A) and day 17 (Fig. 4B), with the expected decrease after oligomycin injection followed by further reduction after rotenone plus AA treatment. Extracellular acidification rate (ECAR) profiles did not reveal apparent group-specific differences at day 10 (Fig. 4C) or day 17 (Fig. 4D), and ECAR increased after BCECs were treated with oligomycin, while ECAR levels were overall lower at day 17.

In comparison with WT_CTRL BCECs, mitoATP production was not impaired in DEL_ADHD and DUP_ADHD BCECs at either day 10 or day 17, with a tendency to increase after prolonged culture within every group, reaching significance only in DUP_ADHD BCECs (Fig. 4E). Compared with WT_CTRL at both day 10 and day 17, DEL_ADHD BCECs showed a lower glycoATP production rate, and prolonged culture induced a significant reduction within every group (Fig. 4F). While total ATP production declined with prolonged culture in every group, a significant decrease was detected only in WT_CTRL and WT_ADHD BCECs (Fig. 4G). After prolonged culture, BCECs exhibited a higher mitoATP and lower glycoATP contribution to total ATP production, reflecting a significant metabolic shift within every group (Fig. 4H). Notably, DEL_ADHD BCECs had significantly increased mitoATP and reduced glycoATP contributions compared with every other group at day 17. Although glycoATP production diminished with prolonged culture, glycolytic reserve was slightly elevated in BCECs at day 17 (Fig. 4I), suggesting that BCECs across all groups retained metabolic capacity to upregulate glycolysis in response to potential impairments in mitochondrial ATP production. In summary, prolonged culture of BCECs promoted a metabolic shift from glycolysis toward OXPHOS, while ADHD *PARK2* CNV-derived BCECs preserved mitochondrial ATP production at both day 10 and day 17.

### Prolonged culture decreases glycolytic lactate abundance and phosphocreatine buffering reserve in BCECs

**Fig. 5:**
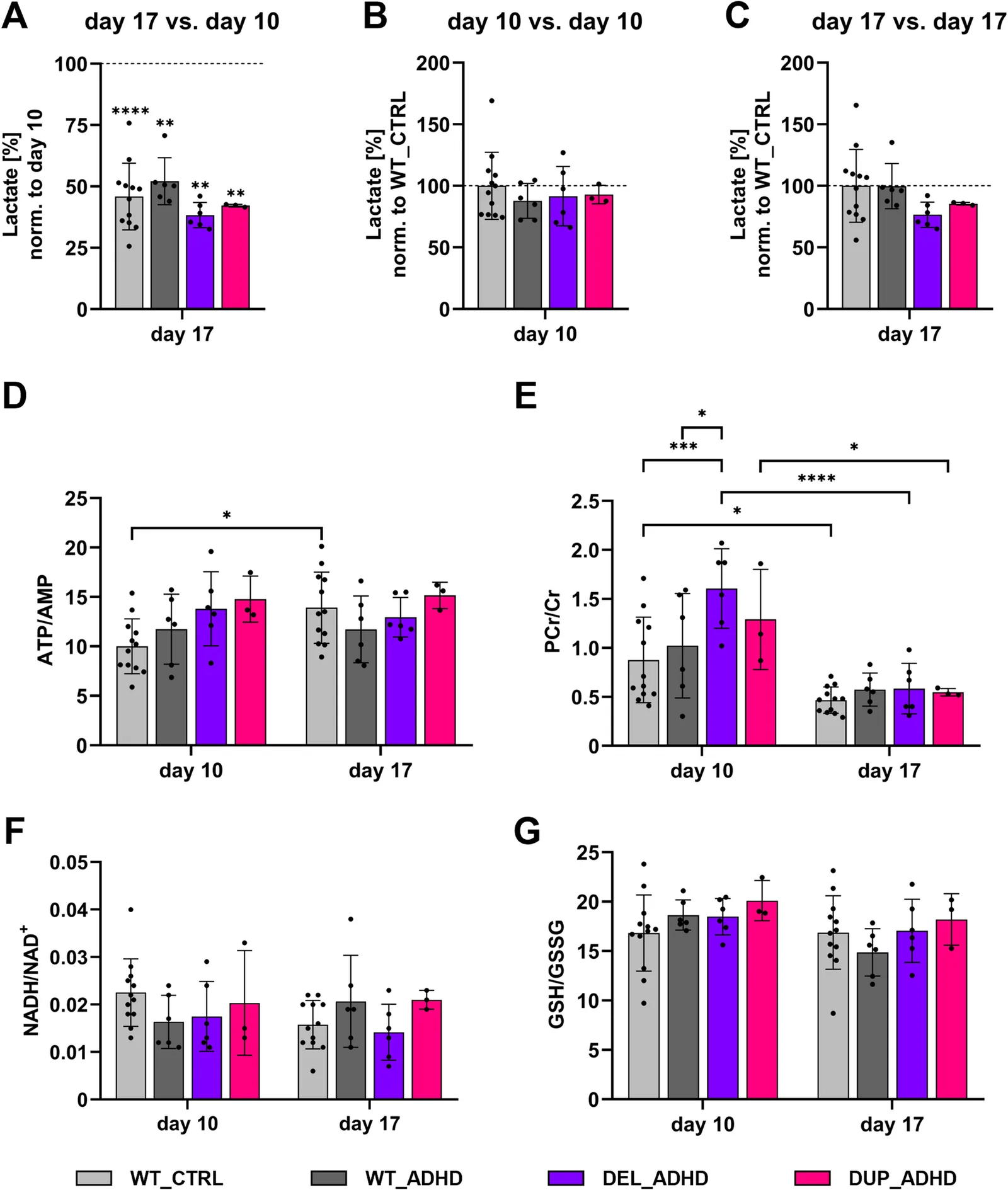
Prolonged culture decreased glycolytic lactate abundance and PCr buffering reserve within BCECs of every group. (**A**–**C**) Lactate levels of BCECs at day 17 normalized to day 10 (**A**), at day 10 normalized to WT_CTRL (**B**), and at day 17 normalized to WT_CTRL (**C**), measured by liquid chromatography-mass spectrometry. Dashed lines indicate respective 100% reference levels. (**D**–**G**) Metabolite ratios of ATP/AMP (**D**), PCr/Cr (**E**), NADH/NAD^+^ (**F**), and GSH/GSSG (**G**) in BCECs at day 10 and day 17. Donor not included: DUP_ADHD (#2). Data are shown as mean ± SD from *n* ≥ 3 independent differentiation experiments, each representing a biological replicate. Statistical analysis was performed using pairwise comparisons by Mann-Whitney or unpaired Welch’s t-tests (**A**), Kruskal-Wallis with Dunn’s post hoc test (**B**, **C**), and two-way ANOVA with Sidak’s post hoc test (**D**–**G**): *p < 0.05, **p < 0.01, ***p < 0.001, ****p < 0.0001

Since ATP production profiles of BCECs changed with culture duration, we quantified intracellular lactate, a major end product of glycolytic metabolism, by targeted metabolomic profiling to validate reduced glycolytic ATP production. As expected, after prolonged culture, lactate abundance was significantly lower in day 17 BCECs compared to day 10 within every group (Fig. 5A). Lactate levels were slightly reduced in DEL_ADHD and DUP_ADHD BCECs compared to WT_CTRL at both day 10 (Fig. 5B) and day 17 (Fig. 5C), although these differences were not statistically significant.

Moreover, to assess broader metabolic disturbance in ADHD *PARK2* CNV-derived BCECs, energy- and redox-related metabolite ratios were determined. In detail, we focused on the following ratios: ATP/AMP (adenosine triphosphate/adenosine monophosphate), PCr/Cr (phosphocreatine/creatine), NADH/NAD^+^ (reduced/oxidized nicotinamide adenine dinucleotide), and GSH/GSSG (reduced/oxidized glutathione), which indicate cellular energy state, phosphate buffering, redox state, and oxidative stress, respectively. ATP/AMP ratio of BCECs did not differ significantly between groups at either day 10 or day 17, while it was significantly higher in WT_CTRL BCECs after prolonged culture (Fig. 5D). At day 10, PCr/Cr ratio was significantly increased in DEL_ADHD BCECs compared to WT_CTRL and WT_ADHD BCECs, with prolonged culture leading to a significant decrease in WT_CTRL, DEL_ADHD, and DUP_ADHD BCECs (Fig. 5E). Ratios of NADH/NAD^+^ (Fig. 5F) and GSH/GSSG (Fig. 5G) were similar across BCECs of all groups at day 10, at day 17, and showed no significant changes after prolonged culture. In conclusion, prolonged culture led to reduced glycolytic lactate abundance and PCr buffering reserve within BCECs of every group, while ADHD *PARK2* CNV-derived BCECs maintained overall cellular energy status and redox balance at both day 10 and day 17.

## Discussion

In this study, hiPSC-derived BCECs were applied to generate BBB in vitro models from individuals with ADHD carrying *PARK2* CNVs, and from *PARK2* wild type adults with and without ADHD, using a total of 10 donor-specific hiPSC lines for group-level comparisons. Given the proposed connection between ADHD, *PARK2* CNVs, mitochondrial dysfunction, and BBB disruption, BCECs were assessed for BBB phenotype, mitochondrial properties, and cellular metabolism. Analyses were performed both at the end of differentiation and after prolonged culture to characterize alterations associated with culture duration.

Independent of ADHD and *PARK2* genotype, BBB in vitro models were successfully established, with BCECs displaying cell type-specific marker expression and physiologically tight barrier function at day 10. In brain endothelial-like cells generated from hiPSCs, extending culture beyond the differentiation endpoint has been associated with a transition toward a more quiescent endothelial state, accompanied by reorganization of junctional marker expression [38, 39], indicating that time in culture is linked to ongoing in vitro phenotypic remodeling. However, in our study, cell type-specific marker expression of BCECs was assessed qualitatively, without apparent culture duration-associated changes. TEER-based barrier integrity of BCECs declined after prolonged culture, with mean values ranging from 1600 to 2500 Ω cm^2^ at day 10 to below 550 Ω cm^2^ at day 17. By contrast, mean NaFl permeability of BCECs remained below 0.5 μm/min at both time points, indicating that lower TEER at day 17 was not accompanied by increased paracellular permeability to the small molecule tracer. The impact of prolonged culture on barrier integrity of hiPSC-derived brain endothelial-like cells has been investigated previously. Hollmann et al. demonstrated sustained barrier function, with TEER remaining above 1000 Ω cm^2^ for at least 8 days [40]. Interestingly, Williams et al. showed that after 7 days of extended culture, TEER changes were heterogeneous in direction across independent differentiation experiments, but mean TEER values were comparable at both time points [38]. Using a closely related differentiation protocol, Choi et al. reported mean TEER of approximately 450 Ω cm^2^ after an additional 7 days of static culture [39], comparable to that of BCECs at day 17 in our study. Evidence from hiPSC-derived brain endothelial-like cells supports a nonlinear relationship between TEER and NaFl permeability, with NaFl permeability reaching a plateau above 500 Ω cm^2^ and remaining similar across culture conditions despite large differences in TEER [41, 42]. Although mean TEER values of BCECs were below 550 Ω cm^2^ at day 17, NaFl permeability was comparable to published mean values of 0.10–0.48 μm/min in other hiPSC-derived brain endothelial-like cells [43–45].

Mitochondrial density in brain endothelial cells is approximately five-fold higher than in peripheral endothelial cells [46]. Nevertheless, it has been reported that brain endothelial cells rely on aerobic glycolysis [47], despite glycolysis being substantially less efficient than OXPHOS in terms of ATP yield per glucose molecule. Glycolysis offers several advantages for endothelial cells, including low oxygen consumption that preserves oxygen availability for perivascular cells, reduced mitochondrial ROS generation while maintaining low-level ROS signaling, and rapid ATP production that supports sprouting and continued vessel growth in avascular hypoxic regions where oxygen becomes limiting sooner than glucose [48, 49]. In this study, hiPSC-derived BCECs generated the majority of ATP via glycolysis at day 10, whereas prolonged culture reduced glycolytic ATP production and increased both absolute mitochondrial ATP production and its relative contribution to total ATP. Despite this redistribution, total ATP production declined after prolonged culture, while glycolytic reserve was slightly higher, suggesting that the reduction in glycolytic ATP production reflected lower utilization of glycolysis rather than diminished glycolytic potential. Consistent with this metabolic shift, intracellular lactate abundance decreased after prolonged culture, further supporting reduced lactate-associated glycolytic metabolism in BCECs at day 17. Similarly, Williams et al. reported lower glycolysis in hiPSC-derived brain endothelial-like cells after 7 days of extended culture [38]. Lactate is not a mere glycolytic end product, as brain endothelial cells actively contribute to lactate homeostasis and monocarboxylate transport across the BBB, and lactate released by the endothelium has been implicated in supporting pericyte function and preserving BBB integrity [50, 51].

Prolonged culture of hiPSC-derived BCECs induced a metabolic shift from glycolysis toward OXPHOS, which coincided with reduced TEER-based barrier integrity. This pattern suggests concurrent, ADHD- and *PARK2* genotype-independent metabolic remodeling associated with culture duration. After prolonged culture, total ATP production rate of BCECs decreased, whereas ATP/AMP ratio was generally maintained or increased, indicating that steady-state cellular energy status was preserved. By contrast, phosphocreatine/creatine ratio was lower after prolonged culture, suggesting a reduction in the immediately available phosphocreatine reserve for rapid ATP regeneration, consistent with the role of creatine kinase and phosphocreatine in linking cellular energy supply to ATP utilization [52, 53]. In a study by Sakamuri et al., passage-associated aging of primary human brain microvascular endothelial cells revealed reduced glycolytic and total ATP production rates, whereas mitochondrial ATP production remained stable from early to pre-senescent passages and decreased only in later senescent cultures [54]. In a separate study using a CD34^+^-derived human BBB model, endothelial senescence coincided with reduced TEER and increased paracellular permeability [55], consistent with broader evidence linking cellular senescence to BBB dysfunction in aging and age-related disease [56]. Given that there are differences between primary human brain microvascular endothelial cells and hiPSC-derived brain endothelial-like cells in barrier function, glycolytic and oxidative metabolism [57], metabolic remodeling with increasing passage in primary cells may reflect different processes than the alterations observed after prolonged culture in differentiated BCECs. Endothelial metabolic remodeling extends beyond glycolysis and OXPHOS and can involve fatty acid, amino acid, and pentose phosphate pathway metabolism, thereby contributing to biosynthesis and redox homeostasis [58]. Although laminar shear stress and blood flow have been shown to maintain oxidative pentose phosphate pathway activity in endothelial cells and thereby support vascular maturation [59], metabolic remodeling was nevertheless observed in BCECs after prolonged static culture, indicating that time in culture can modulate metabolic state even in the absence of flow. After prolonged culture, intracellular ROS levels were overall lower in BCECs, while NADH/NAD^+^ and GSH/GSSG ratios showed no consistent changes. As the GSH/GSSG redox state has been linked to ROS regulation in primary brain endothelial cells [60, 61], the combination of lower ROS levels and a maintained GSH/GSSG ratio in BCECs suggests that prolonged culture did not disrupt the balance between ROS generation and antioxidant defense. Nevertheless, lower ROS levels should not be considered exclusively beneficial, given the role of physiological ROS signaling in BBB homeostasis [62]. Beyond broader signaling implications, lower ROS levels in BCECs coincided with a decrease in ΔΨm after prolonged culture, consistent with evidence that mitochondrial ROS generation is modulated by ΔΨm and can be reduced upon depolarization of the inner mitochondrial membrane [63, 64]. Notably, lower ΔΨm in BCECs was accompanied by an increased mitochondrial ATP production rate after prolonged culture, arguing against the assumption that decreased ΔΨm indicates impaired mitochondrial function. Rather, reduced ΔΨm may reflect greater utilization of the proton-motive force for ATP synthesis relative to proton pumping by the electron transport chain that maintains ΔΨm [65].

Prolonged culture of BCECs was accompanied by broad metabolic remodeling, whereas mitochondrial and metabolic alterations associated with ADHD and *PARK2* CNVs were overall small in comparison. At day 10, mitochondrial count was increased in DUP_ADHD BCECs compared with WT_CTRL, whereas mitochondrial morphology was similar between groups. By contrast, Palladino et al. reported higher aspect ratio and form factor in DUP_ADHD fibroblasts, suggesting a more elongated mitochondrial shape with tubular branching [12]. This discrepancy may reflect cell type-specific differences in mitochondrial morphology, as mitochondrial organization aligns with distinct metabolic and signaling demands across cellular contexts [66]. Although morphology is not a direct measure of mitochondrial function, it remains a useful structural indicator of mitochondrial organization. Higher mitochondrial abundance with stable area per mitochondrion argues against fragmentation and is consistent with enhanced mitochondrial biogenesis [67, 68]. In endothelial cells, peroxisome proliferator-activated receptor γ coactivator 1-α (PGC-1α) links mitochondrial biogenesis to intracellular ROS, as elevated ROS can induce PGC-1α expression while PGC-1α promotes mitochondrial biogenesis and limits ROS accumulation [69, 70]. Accordingly, the greatest mitochondrial abundance in DUP_ADHD BCECs at day 10 may relate to their basal ROS levels, which were also highest among all groups. Enhanced susceptibility to oxidative stress was further indicated by elevated intracellular ROS levels in TBHP-treated DUP_ADHD BCECs at day 10, exceeding those in all other groups. Such a stress-dependent phenotype is consistent with findings by Radtke et al., as the expression of genes related to oxidative stress response, ubiquitin pathways, and mitochondrial quality control showed no baseline differences across donor-specific fibroblasts, hiPSCs, and hiPSC-derived dopaminergic neurons, whereas nutrient deprivation revealed increased expression of the oxidative stress response gene *NQO1* in fibroblasts from ADHD patients carrying *PARK2* CNVs [29]. Under basal conditions, DUP_ADHD BCECs also exhibited reduced TEER-based barrier integrity compared with WT_CTRL at day 10. In brain endothelial cells, elevated oxidative stress can compromise barrier function through altered tight junction protein expression and localization, lower TEER, and increased paracellular permeability [62, 71]. Accordingly, enhanced oxidative stress susceptibility may have contributed to reduced TEER-based barrier integrity of DUP_ADHD BCECs at day 10. At day 17, a group-specific metabolic phenotype was observed in DEL_ADHD BCECs, with mitochondrial ATP production contributing more and glycolytic ATP production less to the total ATP production rate than in all other groups. Despite comparable total ATP production, absolute glycolytic ATP production was lowest in DEL_ADHD BCECs at both time points, demonstrating the least glycolysis-dependent ATP production after prolonged culture. Nevertheless, absolute mitochondrial ATP production remained comparable between groups at day 17, arguing against mitochondrial bioenergetic impairment in DEL_ADHD BCECs.

## Limitations

The inclusion of 10 donor-specific hiPSC lines was a strength of this study, although their division into four different groups resulted in only 2–4 donors per group. Consequently, subtle group-associated effects may have been difficult to distinguish from donor-specific variability and may have remained undetected. Although donor groups were matched for age, sex, and ethnicity, hiPSC lines included in the present study were not isogenic and therefore differed in their broader genetic backgrounds. While genome-editing approaches have been used to generate CNV-corrected isogenic hiPSC lines [72, 73], correction of *PARK2* CNVs could have isolated their contribution within the same donor background. However, as ADHD is a multifactorial and highly polygenic disorder [2, 6], an isogenic control without ADHD cannot be generated through correction of a single genetic variant. Intracellular ROS was assessed using DCFDA, which does not distinguish individual ROS species or their subcellular sources [74]. Therefore, observed differences in intracellular ROS cannot be attributed specifically to mitochondrial ROS production. Nevertheless, DCFDA provides an integrated whole-cell readout of oxidative activity in BCECs. While BCECs were assessed for BBB phenotype, mitochondrial properties, and cellular metabolism only at the end of differentiation (day 10) and after prolonged culture (day 17), onset and progression of the prolonged culture-induced metabolic shift could not be characterized. What this metabolic shift represents biologically remains unclear, as it may reflect adaptation to prolonged static monoculture, reduced proliferation-related metabolic demand, a transition toward quiescence, senescence-associated alterations, progressive deviation from the BBB phenotype, or a combination of these processes. In addition, the present study does not establish whether metabolic remodeling in BCECs contributed to decreased TEER-based barrier integrity, occurred in response to this reduction, or developed independently during prolonged culture, and the mechanisms underlying their potential connection are unresolved.

## Conclusions

Donor-specific BBB in vitro models were successfully established using 10 hiPSC lines across four different groups, with BCECs displaying brain endothelial marker expression, high TEER-based barrier integrity and low NaFl permeability at day 10, demonstrating a robust BBB-associated phenotype. At the end of differentiation, ATP production in BCECs was predominantly glycolytic, consistent with the characteristic reliance of brain endothelial cells on glycolysis [47]. After prolonged culture, NaFl permeability remained low, whereas TEER-based barrier integrity decreased alongside a metabolic shift from glycolysis toward OXPHOS, indicating adverse effects on the maintenance of BBB characteristics. Prolonged culture of BCECs was further accompanied by broad, ADHD- and *PARK2* genotype-independent metabolic remodeling. By comparison, mitochondrial and metabolic alterations associated with ADHD and *PARK2* CNVs were overall small. At day 10, DUP_ADHD BCECs showed greater mitochondrial abundance, the highest basal and oxidative stress-induced intracellular ROS levels, and reduced TEER-based barrier integrity, while at day 17, DEL_ADHD BCECs exhibited the least glycolysis-dependent ATP production profile, although absolute mitochondrial ATP production remained comparable between groups. ADHD- and *PARK2* CNV-related alterations may remain subtle under baseline conditions and become more apparent in response to environmental stressors. Reported associations include perinatal hypoxic-ischemic adversity [75], prenatal undernutrition [76], and maternal infection during pregnancy [77], supporting the investigation of hypoxia, nutrient deprivation, and inflammation as relevant stressors in future experiments. This study identifies culture duration as a critical parameter for the characterization of hiPSC-derived BBB in vitro models and supports the feasibility of using donor-specific BCECs at the end of differentiation to investigate BBB-associated mechanisms in adult ADHD and *PARK2* CNVs.

## Supplementary Information

Below is the link to the electronic supplementary material.

Supplementary Material 1

## Data Availability

The datasets used and/or analyzed during the current study are available from the corresponding author on reasonable request.

## Abbreviations

ΔΨm: Mitochondrial membrane potential
AA: Antimycin A
ADHD: Attention-deficit/hyperactivity disorder
AMP: Adenosine monophosphate
ATP: Adenosine triphosphate
BBB: Blood-brain barrier
BCEC: Brain capillary endothelial-like cell
CDH5: Vascular endothelial cadherin
CLDN5: Claudin-5
CNV: Copy number variation
Cr: Creatine
DCFDA: 2',7'-Dichlorodihydrofluorescein diacetate
DEL_ADHD: *PARK2* deletion with adult ADHD
DUP_ADHD: *PARK2* duplication with adult ADHD
ECAR: Extracellular acidification rate
FCCP: Carbonyl cyanide-p-trifluoromethoxyphenylhydrazone
GLUT1: Glucose transporter 1
GSH: Reduced glutathione
GSSG: Oxidized glutathione
hiPSC: human induced pluripotent stem cell
NAD^+^: Oxidized nicotinamide adenine dinucleotide
NADH: Reduced nicotinamide adenine dinucleotide
NaFl: Sodium fluorescein
OCLN: Occludin
OCR: Oxygen consumption rate
OXPHOS: Oxidative phosphorylation
PC: Permeability coefficient
PCr: Phosphocreatine
PGC-1α: Peroxisome proliferator-activated receptor γ coactivator 1-α
ROS: Reactive oxygen species
TBHP: *Tert*-butyl hydroperoxide
TEER: Transendothelial electrical resistance
TMRM: Tetramethylrhodamine methyl ester
TOM20: Translocase of the outer mitochondrial membrane 20
WT_ADHD: *PARK2* wild type with adult ADHD
WT_CTRL: *PARK2* wild type without adult ADHD

## References

[1] Cecil CAM, Nigg JT. Epigenetics and ADHD: Reflections on Current Knowledge, Research Priorities and Translational Potential. Mol Diagn Ther. 2022;26(6):581–606. 10.1007/s40291-022-00609-y PMC7613776 35933504

[2] Thapar A, Cooper M. Attention deficit hyperactivity disorder. Lancet. 2016;387(10024):1240–50. 10.1016/S0140-6736(15)00238-X 26386541

[3] Faraone SV, et al. Attention-deficit/hyperactivity disorder. Nat Rev Dis Primers. 2024;10(1):11. 10.1038/s41572-024-00495-0 38388701

[4] Polanczyk G, et al. The worldwide prevalence of ADHD: a systematic review and metaregression analysis. Am J Psychiatry. 2007;164(6):942–8. 10.1176/ajp.2007.164.6.942 17541055

[5] Song P, et al. The prevalence of adult attention-deficit hyperactivity disorder: A global systematic review and meta-analysis. J Glob Health. 2021;11:04009. 10.7189/jogh.11.04009 PMC7916320 33692893

[6] Faraone SV, Larsson H. Genetics of attention deficit hyperactivity disorder. Mol Psychiatry. 2019;24(4):562–75. 10.1038/s41380-018-0070-0 PMC6477889 29892054

[7] Demontis D, et al. Discovery of the first genome-wide significant risk loci for attention deficit/hyperactivity disorder. Nat Genet. 2019;51(1):63–75. 10.1038/s41588-018-0269-7 PMC6481311 30478444

[8] Demontis D, et al. Genome-wide analyses of ADHD identify 27 risk loci, refine the genetic architecture and implicate several cognitive domains. Nat Genet. 2023;55(2):198–208. 10.1038/s41588-022-01285-8 PMC10914347 36702997

[9] Gudmundsson OO, et al. Attention-deficit hyperactivity disorder shares copy number variant risk with schizophrenia and autism spectrum disorder. Transl Psychiatry. 2019;9(1):258. 10.1038/s41398-019-0599-y PMC6797719 31624239

[10] Jarick I, et al. Genome-wide analysis of rare copy number variations reveals PARK2 as a candidate gene for attention-deficit/hyperactivity disorder. Mol Psychiatry. 2014;19(1):115–21. 10.1038/mp.2012.161 PMC3873032 23164820

[11] Castelo Rueda MP, et al. Molecular phenotypes of mitochondrial dysfunction in clinically non-manifesting heterozygous PRKN variant carriers. NPJ Parkinsons Dis. 2023;9(1):65. 10.1038/s41531-023-00499-9 PMC10113363 37072441

[12] Palladino VS et al. Energy metabolism disturbances in cell models of PARK2 CNV carriers with ADHD. J Clin Med. 2020;9(12):4092. 10.3390/jcm9124092 PMC7766864 33353000

[13] Pickrell AM, Youle RJ. The roles of PINK1, parkin, and mitochondrial fidelity in Parkinson’s disease. Neuron. 2015;85(2):257–73. 10.1016/j.neuron.2014.12.007 PMC4764997 25611507

[14] Ma K, et al. Mitophagy, Mitochondrial Homeostasis, and Cell Fate. Front Cell Dev Biol. 2020;8:467. 10.3389/fcell.2020.00467 PMC7326955 32671064

[15] Ogutlu H, Kasak M, Tutku Tabur S. Mitochondrial Dysfunction in Attention Deficit Hyperactivity Disorder. Eurasian J Med. 2022;54(Suppl1):187–95. 10.5152/eurasianjmed.2022.22187 PMC11163340 36655466

[16] Corona JC. Role of oxidative stress and neuroinflammation in attention-deficit/hyperactivity disorder. Antioxidants (Basel). 2020;9(11):1039. 10.3390/antiox9111039 PMC7690797 33114154

[17] Archie SR, Shoyaib AA, Cucullo L. Blood-brain barrier dysfunction in CNS disorders and putative therapeutic targets: an overview. Pharmaceutics. 2021;13(11):1779. 10.3390/pharmaceutics13111779 PMC8622070 34834200

[18] Ahirwar LK, et al. Reviewing vascular influences on neuronal migration, cortical development, and neurodevelopmental disorders: focus on autism, ADHD and schizophrenia. Mol Psychiatry. 2025;30(12):5953–66. 10.1038/s41380-025-03200-z PMC12602333 40897863

[19] Uzun N, et al. Investigation of tight junction protein alterations in ADHD: the role of claudin-5, beta-catenin and paxillin. J Atten Disord. 2026;30(6):757–64. 10.1177/10870547251400398 PMC13110292 41358568

[20] Shao Y, et al. Endothelial mitochondria in the blood-brain barrier. Fluids Barriers CNS. 2025;22(1):88. 10.1186/s12987-025-00699-w PMC12376393 40855300

[21] Kim S, Jung UJ, Kim SR. Role of oxidative stress in blood-brain barrier disruption and neurodegenerative diseases. Antioxidants (Basel). 2024;13(12):1462. 10.3390/antiox13121462 PMC11673141 39765790

[22] Kou C, et al. Rnf40 Exacerbates Hypertension-Induced Cerebrovascular Endothelial Barrier Dysfunction by Ubiquitination and Degradation of Parkin. CNS Neurosci Ther. 2025;31(1):e70210. 10.1111/cns.70210 PMC11707429 39777866

[23] Wang Y, et al. Mitochondrial oxidative stress in brain microvascular endothelial cells: Triggering blood-brain barrier disruption. Mitochondrion. 2023;69:71–82. 10.1016/j.mito.2023.01.007 36709855

[24] Brown KA, Samuel S, Patel DR. Pharmacologic management of attention deficit hyperactivity disorder in children and adolescents: a review for practitioners. Transl Pediatr. 2018;7(1):36–47. 10.21037/tp.2017.08.02 PMC5803014 29441281

[25] Nazarova VA, et al. Treatment of ADHD: Drugs, psychological therapies, devices, complementary and alternative methods as well as the trends in clinical trials. Front Pharmacol. 2022;13:1066988. 10.3389/fphar.2022.1066988 PMC9713849 36467081

[26] Ginsberg Y, Quintero J, Anand E, Casillas M, Upadhyaya HP. Underdiagnosis of attention-deficit/hyperactivity disorder in adult patients: a review of the literature. Prim Care Companion CNS Disord. 2014;16(3):PCC.13r01600. 10.4088/PCC.13r01600 PMC4195639 25317367

[27] Philipsen A, et al. Effects of Group Psychotherapy, Individual Counseling, Methylphenidate, and Placebo in the Treatment of Adult Attention-Deficit/Hyperactivity Disorder: A Randomized Clinical Trial. JAMA Psychiatry. 2015;72(12):1199–210. 10.1001/jamapsychiatry.2015.2146 26536057

[28] Kooij JJS, et al. Updated European Consensus Statement on diagnosis and treatment of adult ADHD. Eur Psychiatry. 2019;56:14–34. 10.1016/j.eurpsy.2018.11.001 30453134

[29] Radtke F, et al. ADHD-associated PARK2 copy number variants: A pilot study on gene expression and effects of supplementary deprivation in patient-derived cell lines. Am J Med Genet B Neuropsychiatr Genet. 2022;189(7–8):257–70. 10.1002/ajmg.b.32918 35971782

[30] McNeill RV, et al. Generation of four human induced pluripotent stem cells derived from ADHD patients carrying different genotypes for the risk SNP rs1397547 in the ADHD-associated gene ADGRL3. Stem Cell Res. 2023;67:103016. 10.1016/j.scr.2023.103016 36640473

[31] McNeill RV, et al. hiPSC-derived cortical neurons from ADHD individuals reveal dysregulated glutamatergic development. Mol Psychiatry. 2026;31(2):1041–50. 10.1038/s41380-025-03213-8 PMC12815685 40973785

[32] Lippmann ES, et al. A retinoic acid-enhanced, multicellular human blood-brain barrier model derived from stem cell sources. Sci Rep. 2014;4:4160. 10.1038/srep04160 PMC3932448 24561821

[33] Santa-Maria AR, et al. Transport Studies Using Blood-Brain Barrier In Vitro Models: A Critical Review and Guidelines. Handb Exp Pharmacol. 2022;273:187–204. 10.1007/164_2020_394 33037909

[34] Chaudhry A, Shi R, Luciani DS. A pipeline for multidimensional confocal analysis of mitochondrial morphology, function, and dynamics in pancreatic beta-cells. Am J Physiol Endocrinol Metab. 2020;318(2):E87–101. 10.1152/ajpendo.00457.2019 PMC7052579 31846372

[35] Stringer C, et al. Cellpose: a generalist algorithm for cellular segmentation. Nat Methods. 2021;18(1):100–6. 10.1038/s41592-020-01018-x 33318659

[36] Zink A, et al. High-content screening of mitochondrial polarization in neural cells derived from human pluripotent stem cells. STAR Protoc. 2022;3(3):101602. 10.1016/j.xpro.2022.101602 PMC9361325 35959496

[37] Stirling DR, et al. CellProfiler 4: improvements in speed, utility and usability. BMC Bioinformatics. 2021;22(1):433. 10.1186/s12859-021-04344-9 PMC8431850 34507520

[38] Williams LM, et al. Prolonged culturing of iPSC-derived brain endothelial-like cells is associated with quiescence, downregulation of glycolysis, and resistance to disruption by an Alzheimer’s brain milieu. Fluids Barriers CNS. 2022;19(1):10. 10.1186/s12987-022-00307-1 PMC8817611 35123529

[39] Choi J, Mathew S, Oerter S, Appelt-Menzel A, Hansmann J, Schmitz T. Online measurement system for dynamic flow bioreactors to study barrier integrity of hiPSC-based blood-brain barrier in vitro models. Bioengineering (Basel). 2022;9(1):39. 10.3390/bioengineering9010039 PMC8773345 35049748

[40] Hollmann EK, et al. Accelerated differentiation of human induced pluripotent stem cells to blood-brain barrier endothelial cells. Fluids Barriers CNS. 2017;14(1):9. 10.1186/s12987-017-0059-0 PMC5390351 28407791

[41] Mantle JL, Min L, Lee KH. Minimum Transendothelial Electrical Resistance Thresholds for the Study of Small and Large Molecule Drug Transport in a Human in Vitro Blood-Brain Barrier Model. Mol Pharm. 2016;13(12):4191–8. 10.1021/acs.molpharmaceut.6b00818 27934481

[42] Neal EH, et al. Influence of basal media composition on barrier fidelity within human pluripotent stem cell-derived blood-brain barrier models. J Neurochem. 2021;159(6):980–91. 10.1111/jnc.15532 PMC8688328 34716922

[43] Canfield SG, et al. An isogenic blood-brain barrier model comprising brain endothelial cells, astrocytes, and neurons derived from human induced pluripotent stem cells. J Neurochem. 2017;140(6):874–88. 10.1111/jnc.13923 PMC5339046 27935037

[44] Danz K, Höcherl T, Wien SL, Wien L, von Briesen H, Wagner S. Experimental comparison of primary and hiPS-based in vitro blood-brain barrier models for pharmacological research. Pharmaceutics. 2022;14(4):737. 10.3390/pharmaceutics14040737 PMC9031459 35456571

[45] Koenig L, Ramme AP, Faust D, Mayer M, Flötke T, Gerhartl A, et al. A human stem cell-derived brain-liver chip for assessing blood-brain-barrier permeation of pharmaceutical drugs. Cells. 2022;11(20):3295. 10.3390/cells11203295 PMC9600760 36291161

[46] Bayir E, Sendemir A. Role of intermediate filaments in blood-brain barrier in health and disease. Cells. 2021;10(6):1400. 10.3390/cells10061400 PMC8226756 34198868

[47] Kim ES, et al. Brain Endothelial Cells Utilize Glycolysis for the Maintenance of the Transcellular Permeability. Mol Neurobiol. 2022;59(7):4315–33. 10.1007/s12035-022-02778-7 35508867

[48] Leung SWS, Shi Y. The glycolytic process in endothelial cells and its implications. Acta Pharmacol Sin. 2022;43(2):251–9. 10.1038/s41401-021-00647-y PMC8791959 33850277

[49] Li X, Sun X, Carmeliet P. Hallmarks Endothelial Cell Metabolism Health Disease Cell Metab. 2019;30(3):414–33. 10.1016/j.cmet.2019.08.011 31484054

[50] Wang J, et al. Brain Endothelial Cells Maintain Lactate Homeostasis and Control Adult Hippocampal Neurogenesis. Cell Stem Cell. 2019;25(6):754–e7679. 10.1016/j.stem.2019.09.009 31761722

[51] Lee HW, et al. Endothelium-derived lactate is required for pericyte function and blood-brain barrier maintenance. EMBO J. 2022;41(9):e109890. 10.15252/embj.2021109890 PMC9058541 35243676

[52] Wallimann T, Tokarska-Schlattner M, Schlattner U. The creatine kinase system and pleiotropic effects of creatine. Amino Acids. 2011;40(5):1271–96. 10.1007/s00726-011-0877-3 PMC3080659 21448658

[53] Dringen R, et al. ATP Metabolism of Astrocytes: Consumption, Regeneration and Restoration. Neurochem Res. 2025;50(6):357. 10.1007/s11064-025-04604-7 PMC12612006 41222868

[54] Sakamuri S, et al. Glycolytic and Oxidative Phosphorylation Defects Precede the Development of Senescence in Primary Human Brain Microvascular Endothelial Cells. Geroscience. 2022;44(4):1975–94. 10.1007/s11357-022-00550-2 PMC9616994 35378718

[55] Novo JP, et al. Blood-brain barrier dysfunction in aging is mediated by brain endothelial senescence. Aging Cell. 2024;23(9):e14270. 10.1111/acel.14270 PMC11488312 39143890

[56] Knopp RC, et al. Cellular senescence and the blood-brain barrier: Implications for aging and age-related diseases. Exp Biol Med (Maywood). 2023;248(5):399–411. 10.1177/15353702231157917 PMC10281623 37012666

[57] Weber CM, et al. Induced pluripotent stem cell-derived cells model brain microvascular endothelial cell glucose metabolism. Fluids Barriers CNS. 2022;19(1):98. 10.1186/s12987-022-00395-z PMC9733016 36494870

[58] Falkenberg KD, et al. The metabolic engine of endothelial cells. Nat Metab. 2019;1(10):937–46. 10.1038/s42255-019-0117-9 32694836

[59] Facchinello N, et al. Oxidative pentose phosphate pathway controls vascular mural cell coverage by regulating extracellular matrix composition. Nat Metab. 2022;4(1):123–40. 10.1038/s42255-021-00514-4 PMC7612297 35102339

[60] Sivandzade F, Alqahtani F, Cucullo L. Impact of chronic smoking on traumatic brain microvascular injury: An in vitro study. J Cell Mol Med. 2021;25(15):7122–34. 10.1111/jcmm.16741 PMC8335687 34160882

[61] Gao M, Fu J, Wang Y. The lncRNA FAL1 protects against hypoxia-reoxygenation- induced brain endothelial damages through regulating PAK1. J Bioenerg Biomembr. 2020;52(1):17–25. 10.1007/s10863-019-09819-2 31927658

[62] Shuvalova M, et al. The role of reactive oxygen species in the regulation of the blood-brain barrier. Tissue Barriers. 2025;13(2):2361202. 10.1080/21688370.2024.2361202 PMC12363512 38808582

[63] Chenna S, et al. Mechanisms and mathematical modeling of ROS production by the mitochondrial electron transport chain. Am J Physiol Cell Physiol. 2022;323(1):C69–83. 10.1152/ajpcell.00455.2021 35613354

[64] Vyssokikh MY, et al. Mild depolarization of the inner mitochondrial membrane is a crucial component of an anti-aging program. Proc Natl Acad Sci U S A. 2020;117(12):6491–501. 10.1073/pnas.1916414117 PMC7104298 32152094

[65] Tovar-Ferrero O, et al. Measuring mitochondrial membrane potential. EMBO J. 2025;44(24):7334–45. 10.1038/s44318-025-00632-9 PMC12706018 41249505

[66] Glancy B, et al. The Functional Impact of Mitochondrial Structure Across Subcellular Scales. Front Physiol. 2020;11:541040. 10.3389/fphys.2020.541040 PMC7686514 33262702

[67] Coronado M, et al. Physiological Mitochondrial Fragmentation Is a Normal Cardiac Adaptation to Increased Energy Demand. Circ Res. 2018;122(2):282–95. 10.1161/CIRCRESAHA.117.310725 PMC5775047 29233845

[68] Wang C, et al. Serine synthesis controls mitochondrial biogenesis in macrophages. Sci Adv. 2024;10(20):eadn2867. 10.1126/sciadv.adn2867 PMC11100566 38758794

[69] Valle I, et al. PGC-1alpha regulates the mitochondrial antioxidant defense system in vascular endothelial cells. Cardiovasc Res. 2005;66(3):562–73. 10.1016/j.cardiores.2005.01.026 15914121

[70] Sawada N, et al. Endothelial PGC-1alpha mediates vascular dysfunction in diabetes. Cell Metab. 2014;19(2):246–58. 10.1016/j.cmet.2013.12.014 PMC4040246 24506866

[71] Vagvolgyi M, et al. 17-Oxime ethers of oxidized ecdysteroid derivatives modulate oxidative stress in human brain endothelial cells and dose-dependently might protect or damage the blood-brain barrier. PLoS ONE. 2024;19(2):e0290526. 10.1371/journal.pone.0290526 PMC10883584 38386637

[72] Munezane H, et al. Elimination of the extra chromosome of Dup15q syndrome iPSCs for cellular and molecular investigation. Eur J Cell Biol. 2024;103(3):151446. 10.1016/j.ejcb.2024.151446 39059105

[73] Ye T, et al. Efficient manipulation of gene dosage in human iPSCs using CRISPR/Cas9 nickases. Commun Biol. 2021;4(1):195. 10.1038/s42003-021-01722-0 PMC7881037 33580208

[74] Kalyanaraman B, et al. Measuring reactive oxygen and nitrogen species with fluorescent probes: challenges and limitations. Free Radic Biol Med. 2012;52(1):1–6. 10.1016/j.freeradbiomed.2011.09.030 PMC3911769 22027063

[75] Zhu T, et al. Association Between Perinatal Hypoxic-Ischemic Conditions and Attention-Deficit/Hyperactivity Disorder: A Meta-Analysis. J Child Neurol. 2016;31(10):1235–44. 10.1177/0883073816650039 27229008

[76] Cortés-Albornoz MC, García-Guáqueta DP, Velez-van-Meerbeke A, Talero-Gutiérrez C. Maternal nutrition and neurodevelopment: a scoping review. Nutrients. 2021;13(10):3530. 10.3390/nu13103530 PMC8538181 34684531

[77] Ayubi E, Mansori K. Maternal Infection during Pregnancy and Attention-Deficit Hyperactivity Disorder in Children: A Systematic Review and Meta-Analysis. Iran J Public Health. 2022;51(12):2674–87. 10.18502/ijph.v51i12.11458 PMC9874197 36742242

