# Supplementary material for: Adult ADHD, PARK2 copy number variations and the blood-brain barrier: Culture duration-dependent metabolic alterations in human induced pluripotent stem cell-derived in vitro models
Source: Fluids Barriers CNS. 2026 Oct 24;23:123. doi: 10.1186/s12987-026-00892-5 (PMC13715133; doi:10.1186/s12987-026-00892-5)
Supplement: Supplementary file 1 [file 12987_2026_892_MOESM1_ESM.docx]

Supporting Information

Adult ADHD, *PARK2* copy number variations and the blood-brain barrier: Metabolic studies in human induced pluripotent stem cell-derived in vitro models

Markus Glaser, Liane Dieterich, Laura Ceslik, Zora Schickardt, Sabine Gätzner, Luisa Schmitt, Heike Oberwinkler, Jasmin Reining, Mike Friedrich, Katherina Hemmen, Werner Schmitz, Rhiannon Victoria McNeill, Sarah Kittel-Schneider, Antje Appelt-Menzel*

**Figure S1**. Validation of donor-specific hiPSC line quality through pluripotency, trilineage differentiation, and Sendai virus clearance. (A–B) Representative immunofluorescence images of reprogrammed hiPSC lines WT_ADHD (#1) and DUP_ADHD (#2). HiPSCs were stained for pluripotency markers (A) SSEA4 (green), OCT4 (red), and TRA-1-60 (green) and for trilineage differentiation markers (B) TUBB3 (red), SMA (orange) and AFP (green), followed by DAPI counterstaining (blue). Scale bars, 200 µm. (C) RT-PCR analysis of pluripotency-associated transcripts *OCT4*, *SOX2*, *NANOG*, and *DPPA5* and Sendai vector-related transcripts *c-MYC*, *KLF4*, *KOS*, and *SeV* in hiPSC lines WT_ADHD (#1) and DUP_ADHD (#2), with *GAPDH* as control.

**Table S1**. Donor and hiPSC line characteristics of the 10 donor-specific hiPSC lines used in this study.

| **Donor group** | ***PARK2* genotype** | **Diagnosis** | **Age** | **Sex** | **Ethnicity** | **Reference** |
| --- | --- | --- | --- | --- | --- | --- |
| **WT_CTRL** |  |  |  |  |  |  |
| #1 | Wild type | CTRL | 27 | Female | Caucasian | [12, 27] |
| #2 | Wild type | CTRL | 42 | Female | Caucasian | [12, 27] |
| #3 | Wild type | CTRL | 28 | Male | Caucasian | [29] |
| #4 | Wild type | CTRL | 46 | Male | Caucasian | [29] |
|  |  |  |  |  |  |  |
| **WT_ADHD** |  |  |  |  |  |  |
| #1 | Wild type | ADHD | 46 | Male | Caucasian | Figure S2 |
| #2 | Wild type | ADHD | 39 | Male | Caucasian | [28] |
|  |  |  |  |  |  |  |
| **DE**L**_ADHD** |  |  |  |  |  |  |
| #1 | Deletion | ADHD | 43 | Female | Caucasian | [12, 27] |
| #2 | Deletion | ADHD | 47 | Male | Caucasian | [12, 27] |
|  |  |  |  |  |  |  |
| **DUP_ADHD** |  |  |  |  |  |  |
| #1 | Duplication | ADHD | 28 | Female | Caucasian | [12, 27] |
| #2 | Duplication | ADHD | 48 | Female | Caucasian | Figure S2 |

**Figure S2**. ADHD *PARK2* CNV-derived BCECs preserved mitochondrial morphology. (A–B) Representative immunofluorescence images of BCECs at day 10 (A) and day 17 (B) derived from donor-specific hiPSC lines WT_CTRL (#1), WT_ADHD (#1), DEL_ADHD (#1), and DUP_ADHD (#1). BCECs were stained for TOM20 (green), followed by DAPI counterstaining (blue). Scale bars, 20 µm. Segmentation masks were generated using Mitochondria Analyzer.
